# Sarcoidosis with Severe Bone Involvement: A Case Report and Literature Review

**DOI:** 10.3390/diagnostics13182990

**Published:** 2023-09-18

**Authors:** Haoshen Feng, Jiangwei Ma, Yabin Zhao, Rui Zheng, Wei Wang

**Affiliations:** 1 Department of Pulmonary and Critical Care Medicine, Shengjing Hospital of China Medical University, Shenyang 110004, China; 2 Department of Pulmonary and Critical Care Medicine, The First Hospital of China Medical University, Shenyang 110001, China

**Keywords:** sarcoidosis, bone, sinonasal sarcoidosis, granulomatous disease

## Abstract

Sarcoidosis is a systemic granulomatous disease of the lungs that commonly involves intrathoracic lymph nodes. Here, we report the case of a 68-year-old woman who complained of shortness of breath and had suffered from the enlargement of intrathoracic lymph nodes for 12 years, swelling of the right middle finger for 7 years, and nasal obstruction for 2 years. The damage to the phalange was aggravated continuously and a malignant lesion could not be excluded, thus leading to amputation of the right middle finger. Pathological data indicated chronic inflammatory granulomatous disease and anti-acid staining was negative. Examination of the paranasal sinuses indicated destruction of the sinonasal bone and the swollen mucosa. Combined with the elevated ratio of CD4+/CD8+ T cells in bronchoalveolar lavage fluid and other results, the patient was finally diagnosed with sarcoidosis and received corticosteroid therapy. The shortness of breath and abnormality of the nose were significantly improved after treatment. Our case demonstrated the process of differential diagnosis for systemic granulomatous diseases, indicating the necessity of corticosteroid therapy for systematic sarcoidosis.

## 1. Introduction

Sarcoidosis is a multisystemic granulomatous disease with an indetermined etiology [1]. Some experts believe that sarcoidosis is caused by the human immune system reacting to unknown substances (infectious pathogens, chemicals or dust, etc.) in genetically susceptible people [2]. The diagnosis of sarcoidosis mainly depends on the pathological manifestations of granuloma, which consist of multinucleated giant cells at the core and peripheral immunocytes [3]. The lungs and intrathoracic lymph nodes are commonly involved, although other organs can be affected, including the skin, eyes, bone and heart [4]. The incidence rate of extrathoracic sarcoidosis is relatively lower than that of intrathoracic sarcoidosis. For example, the frequency of bone involvement was between 3% and 13% [5], and the frequency of sinonasal involvement is reported to be 1–6.5% [6,7]. Here, we report a rare case of a patient with sarcoidosis with severe bone Involvement. In this case, the patient mainly experienced involvement of the phalange bones for 7 years, which ultimately led to finger amputation, accompanied by ocular disorder, lymph node enlargement and sinonasal involvement. The highlight of this case was the long course of severe bone involvement without significant intrapulmonary manifestations. 

## 2. Case Presentation

**Figure 1:**
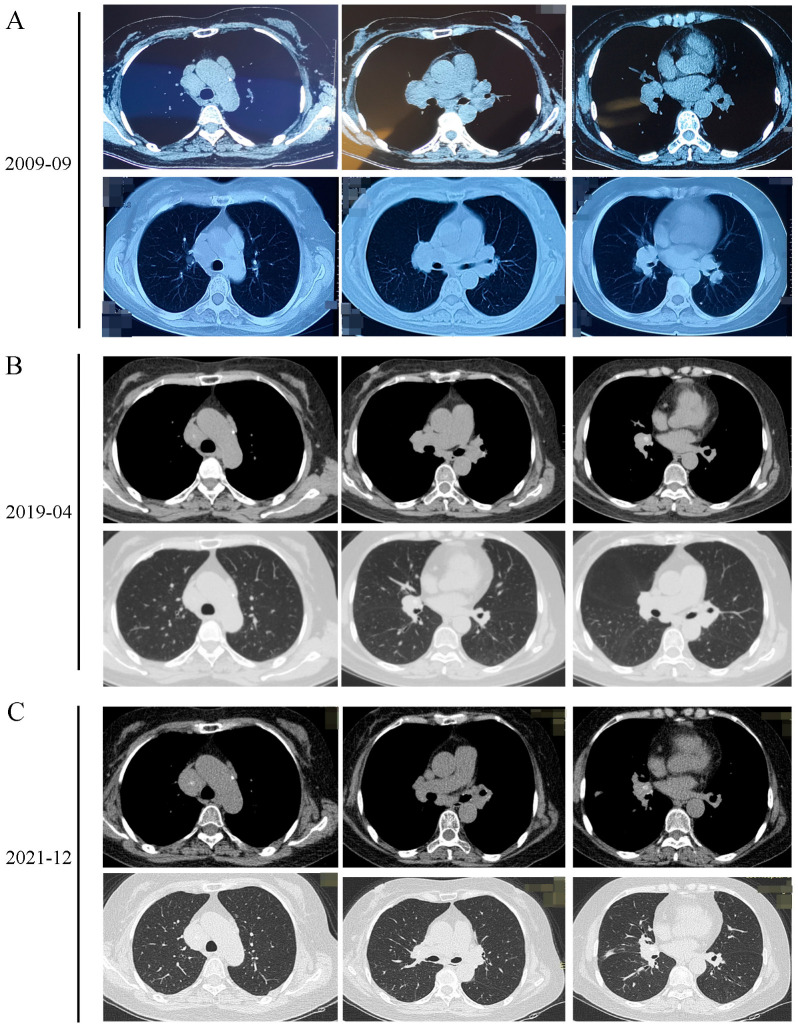
(**A**) The enlargement of intrathoracic lymph nodes was detected by chest CT scans in 19 September 2009. (**B**) 3 April 2019. (**C**) 2 December 2021. A new calcification of the mediastinal lymph nodes and a new opacity in the right middle lobe of the lung appeared in 3 April 2019 and 2 December 2021.

A 68-year-old woman was admitted with a 12-year history of enlargement of the intrathoracic lymph nodes and a 2-year history of shortness of breath. In 2009, enlargement of her intrathoracic lymph nodes was determined by chest computed tomography (CT) and physical examination (Figure 1A); however, she did not receive any treatment due to a lack of symptoms. In 2019, the patient experienced progressive shortness of breath during physical activities; this was occasionally accompanied by chest pain. The CT scan performed in 2019 demonstrated a new calcification of mediastinal lymph nodes, and the reduced size of lymph nodes compared with the previous examination (Figure 1B). The patient received intermittent oral administration of Andrographis Paniculate and the inhalation of corticosteroids when the shortness of breath worsened. She was admitted to hospital in December 2021 due to the exacerbation of breath shortness and underwent a chest CT scan, which indicated an enlargement of the intrathoracic lymph nodes with scattered calcification and a new opacity in the right middle lobe of the lung (Figure 1C). The patient had undergone appendectomy 30 years earlier and had a history of coronary heart disease for more than 10 years without being fully examined and treated. Laboratory test results are shown in Table 1. The patient had normal pulmonary function (forced vital capacity (FVC)%: 100.2%, forced expiratory volume in one second (FEV1)%: 92.5% and FEV1/FVC: 76.69%). The results of echocardiography indicated normal systolic function of the left ventricle (ejection fraction: 62%) and degeneration of the aortic valve. Ultrasound examination received fatty liver and lymph nodes in the neck, subclavian, armpit and groin.

**Figure 2:**
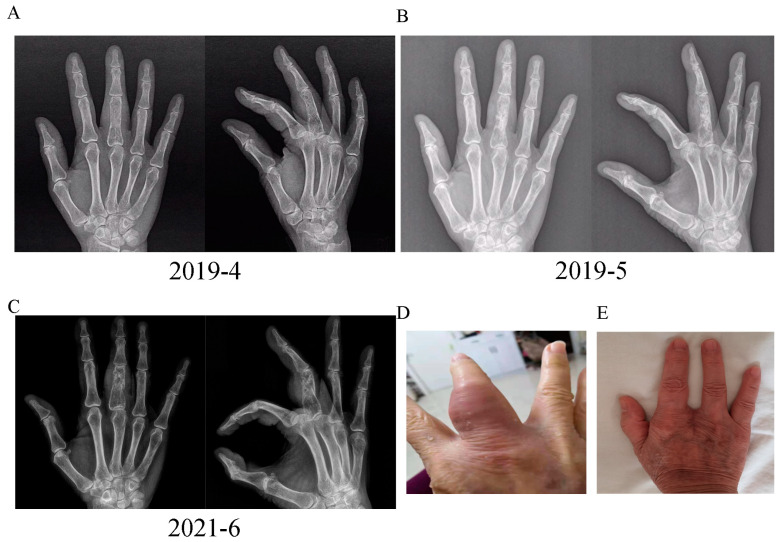
(**A**) Digital X-ray examination indicated ground glass opacity in the bone marrow cavity and destruction of the bone cortex in the right proximal middle phalanx in 3 April 2019. (**B**) Digital X-ray examination was performed in 22 May 2019; this indicated high density opacity which was the bone graft in the right proximal middle phalanx. (**C**) Digital X-ray examination indicated hyperplastic and pathological fracture-like manifestations in the right proximal middle phalanx. (**D**) The swollen middle phalanx of the right hand prior to amputation surgery. (**E**) The right hand after amputation surgery.

**Figure 3:**
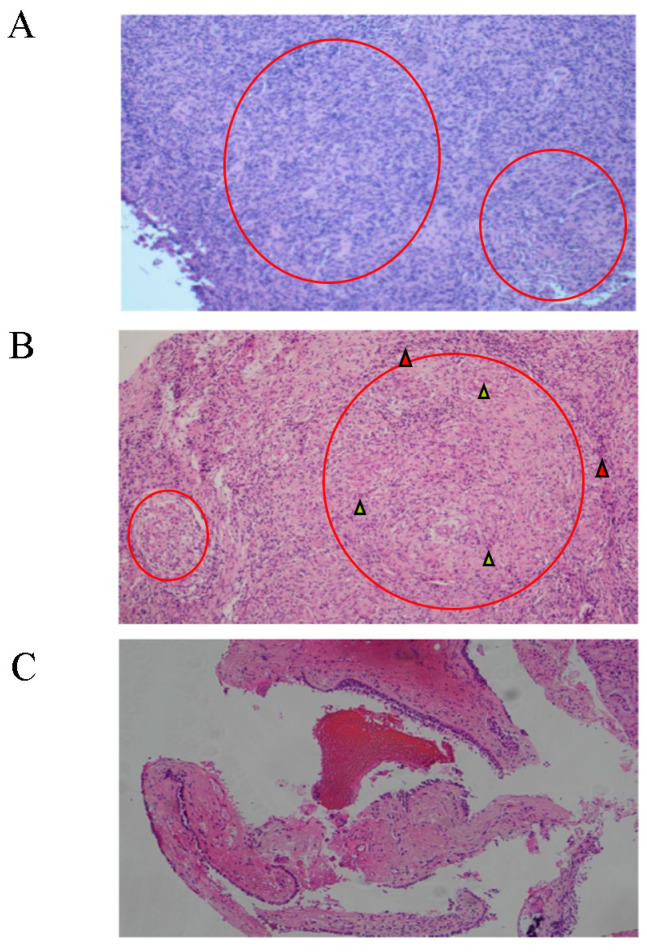
Pathological results for the right proximal middle phalanx indicated chronic inflammatory granulomas in 2019 (**A**) and 2021 (**B**). Red circles showed the position of granulomas. Yellow arrows showed the multinucleated giant cells and red arrows showed the infiltrated lymphocytes. Pathological analysis of mediastinal lymph nodes indicated cellulose with lymphocyte infiltration (**C**).

**Figure 4:**
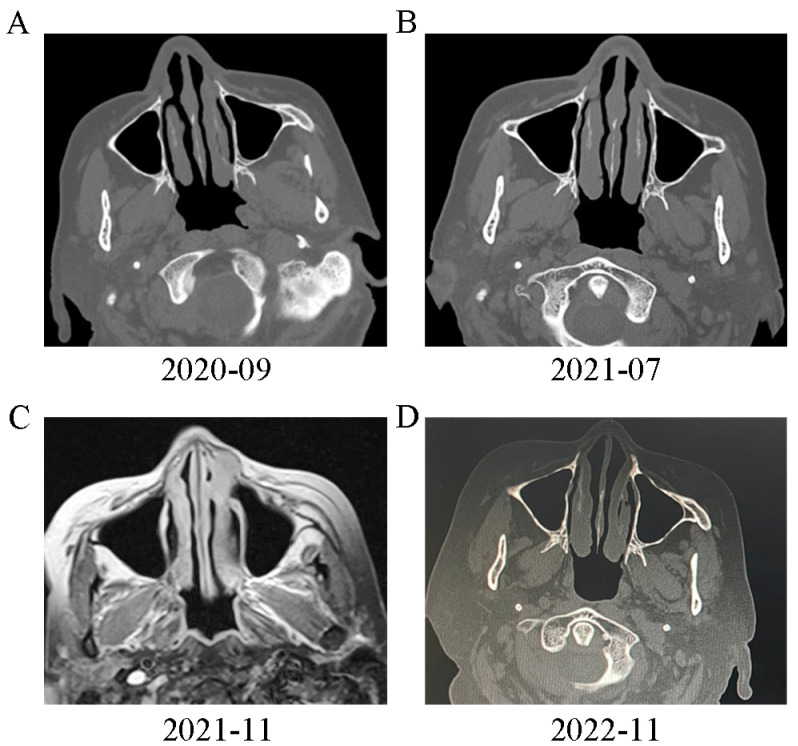
(**A**) Sinonasal CT in 7 September 2020 revealed irregularities in the left sinonasal bone with multiple low-density areas and the local swollen soft tissues. (**B**) The sinonasal CT in 29 July 2021 indicated enlargement of the destruction area in sinonasal bone with both sides affected. (**C**) Sinonasal magnetic resonance imaging in 5 November 2021 indicated local narrowing of the left nasal meatus in addition to the above manifestations. (**D**) After prednisone treatment for 3 months, sinonasal CT was performed and indicated significant improvement of destruction in the sinonasal bone.

We also observed that the patient also had a series of extrapulmonary abnormalities. First, the patient’s right middle finger became slightly swollen in 2015, although she did not seek medical advice at this time. In April 2019, the patient felt pain and experienced an exacerbation of swelling in her right middle finger; furthermore, some soft tissue nodules also appeared in her right arm. Digital X-ray examination of the right hand indicated ground glass opacity in the bone marrow cavity and destruction of the bone cortex (Figure 2A). The patient underwent curettage and bone grafting of the proximal phalanx of the right middle finger. A soft jelly-like substance was removed and pathological analysis suggested granulomas with infiltration of lymphocytes and plasma cells without caseous necrosis (Figure 3A). A digital X-ray examination was performed after surgery (Figure 2B), and the symptoms of the right hand were partially relieved. In 2021, the patient’s right middle finger became swollen and painful again (Figure 2D); digital X-ray examination indicated hyperplastic and pathological fracture-like manifestations in the proximal phalanx of the right middle finger (Figure 2C). Considering the possibility of malignant lesions, the patient’s finger was amputated (Figure 2E); pathological analysis of the right middle finger and right arm tissue indicated chronic inflammatory granulomas without caseous necrosis (Figure 3B). In addition, the patient had been diagnosed with ocular tuberculosis 50 years earlier and had been treated by isoniazid. She was diagnosed with binocular angle-closure glaucoma and age-related cataract in November 2020. Subsequently, the patient suffered from nasal obstructive for two years, and underwent sinonasal CT examination in September 2020; this revealed irregularities in the left sinonasal bone with multiple low-density areas and local swollen soft tissues (Figure 4A). The patient did not receive any treatment for this. The nasal obstruction became aggravated, and the tissues around the nose gradually became swollen. A sinonasal CT in July 2021 indicated enlargement of the destruction area in sinonasal bone with both sides affected (Figure 4B). Sinonasal magnetic resonance imaging in November 2021 indicated local narrowing of the left nasal meatus in addition to the other manifestations noted previously (Figure 4C). 

We performed fiberoptic bronchoscopy on the patient; no obvious abnormality was detected. The right middle lobe was performed with bronchoalveolar lavage (BAL); there were 0.14 × 10^6^ cells per milliliter in the BAL fluid. The proportions of macrophages, neutrophils, lymphocytes and eosinophils were 63%, 1%, 36% and 0 in BAL fluid, respectively. The counts of CD4+ and CD8+ T cells were 100 and 8 per microliter in BAL fluid, and the ratio of CD4+/CD8+ T cells was 12.5. GeneXpert analysis of the BAL fluid was negative. The biopsy of lung tissues and mediastinal lymph nodes was performed by endobronchial ultrasound-guided transbronchial needle aspiration (EBUS-TBNA), and the pathological results indicated chronic inflammatory infiltration accompanied by fibrosis in lung tissues and cellulose in the lymph nodes (Figure 3C). 

**Figure 5:**
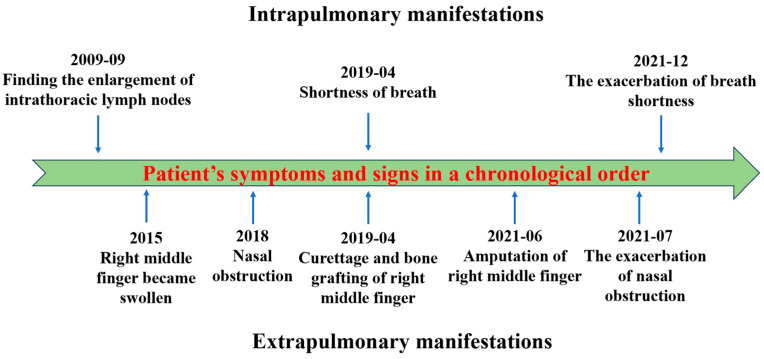
The patient’s manifestations were exhibited in a chronological order.

Differential diagnosis was mainly focused on granulomatous diseases, which could be primary divided into infectious ones and non-infectious ones according to the etiology. For infectious granulomatous diseases, tuberculosis and fungal infections are the most common causes. The pure protein derivatives (PPD) and T-SPOT test was negative (Table 1), and anti-acid staining of biopsies was also negative; this could exclude the possibility of tuberculosis. The Glucatell test, glactomannan test and cryptococcus antigen test were also performed; these were all negative (Table 1). Thus, the possibility of fungal infection was very low. Non-infectious granulomatous diseases include sarcoidosis, hypersensitivity pneumonia, vasculitis, connective tissue disease (CTD) and related diseases. The patient had no CTD-related symptoms or allergen exposure experience, and her CTD-related antibodies were all negative (Table 1). Granulomatous vasculitis (eosinophilic granulomatosis with polyangiitis (EGPA) and granulomatosis with polyangiitis (GPA)) may lead to manifestations like our patient in the case. Our patient only has sinusitis and lung infiltration imaging, which do not support the diagnosis of EGPA. The negative anti-neutrophil cytoplasmic antibodies, normal urinalysis (Table 1) and the lack of necrosis in granulomas exclude the possibility of GPA in our patient. The IgG4 staining of biopsies was also negative. Considering the systematic granuloma manifestations and elevated CD4+/CD8+ T cells in BAL fluid, the patient was finally diagnosed with sarcoidosis. The patient was suggested to start oral corticosteroid therapy, but she temporarily refused the therapy due to adverse reactions. In August 2022, the patient received prednisone treatment at a dose of 30 mg per day due to the exacerbation of breath shortness. The dose of corticosteroid was tapered by 5 mg/day every month (30 mg/day for the first month, 25 mg/day for the second month and 20 mg/day for the third month). After prednisone treatment for 3 months, the breath shortness was largely ameliorated, and the sinonasal CT indicated significant improvement of destruction in the sinonasal bone (Figure 4D). To present the patient’s symptoms and signs better, Figure 5 summarizes the patient’s manifestations in a chronological order.

## 3. Discussion

This paper reports a case of sarcoidosis with severe bone involvement occurring over several years. In this case, the patient experienced swelling of right middle finger, which was suspected as a malignant disease and led to finger amputation. At the same time, the patient also experienced enlargement of the intrathoracic lymph nodes, ocular disorder and sinonasal involvement. Excluding other potential diseases, the patient was finally diagnosed with sarcoidosis according to the granulomatous manifestations in the finger and arm tissues. The strength of our case was manifested by long-term severe bone involvement without a significant intrapulmonary lesion; however, our case was limited by the lack of sinonasal pathological data. Furthermore, whether the ocular abnormality was caused by sarcoidosis could not be fully determined.

Sarcoidosis is the most common non-infectious systemic granulomatous disease and commonly involves the lungs and intrathoracic lymph nodes. Multiple granulomatous manifestations were the critical characteristics of our patient, and the tissues of the right middle finger and arm exhibited typical non-caseous necrotizing granuloma. Although no granuloma was detected in the pathological results of lung tissues and intrathoracic lymph nodes. However, we could still not exclude the existence of a granuloma in the lung tissues and intrathoracic lymph nodes for the following reasons: (1) the tissues acquired by EBUS-TBNA were too small to represent the whole lesion completely, and (2) the granuloma may have transferred fibrotic tissues during the development of sarcoidosis. However, some other systemic granulomatous diseases needed to be excluded. First, tuberculosis is characterized by caseous necrotizing granuloma. Osteoarticular tuberculosis accounts for 15% of all cases of extrapulmonary tuberculosis [8], and mainly occurs in the spine and the load-bearing joints [9]. Pain, fever, localized swelling, fistulas and drainage are all common symptoms of osteoarticular tuberculosis [10], and some patients even experience bone destruction and cold abscesses [11]. Although our patient had experienced swelling in the right middle finger for several years, neither pyogenic necrosis nor fistulas were evident. Combined with the negative results of anti-acid staining and other clinical information, we excluded the possibility of tuberculosis infection. Granulomatous vasculitis (EGPA and GPA) may lead to manifestations like our patient in the case. The diagnostic criteria of EGPA should meet at least four following items: asthma symptoms, the percentages of peripheral blood eosinophils > 10%, sinusitis, lung infiltration imaging, vasculitis with eosinophilia infiltration in pathology and polyneuropathy [12]. Our patient only has sinusitis and lung infiltration imaging, which do not support the diagnosis of EGPA. Patients with GPA could have systematic granuloma formation with sinus and lung involvement, and the granulomas of GPA are usually accompanied by necrosis [13]. However, the negative anti-neutrophil cytoplasmic antibodies, normal urinalysis and the lack of necrosis in granulomas exclude the possibility of GPA in our patient. Besides, lethal midline granuloma is a clinical–pathological entity characterized by swelling, ulceration, necrosis and destruction of the central face, with pathologic features of inflammation and granulation [14]. Although our patient has sinus granuloma formation, the lack of necrosis and destruction of the central face indicates little possibility of lethal midline granuloma in our case. Furthermore, IgG4-related disease mainly manifests as multiple swollen organs and could also lead to granuloma [15]; however, we did not detect any positive results for IgG4 in blood and biopsy tests. 

Bone lesions occurring with sarcoidosis were relatively rare compared with the involvement of other organs. The characteristics of bone sarcoidosis in previous retrospective studies are shown in Table 2 [16,17,18,19,20,21,22]. The reason for bone sarcoidosis was not determined. Although the elevation of serum 1,25(OH)2D3 level was believed to participate in the bone abnormalities of some patients with sarcoidosis [23], we believed that the bone destruction in our patient was not caused by this factor due to the normal levels of calcium in the serum and urine. We speculated that the bone damage in our patient may have resulted from the osteoclastic reaction caused by granulomas, which was mediated by the following means: (1) the sarcoidosis granulomas increased the local level of 1,25(OH)2D3 by promoting the conversion of Vit D-25 to Vit D-1,25 or directly releasing Vit D-1,25 [24,25], and the high level of 1,25(OH)2D3 promoted bone resorption by stimulating osteoclastic activity, and (2) the granuloma may have secreted substance, for example osteoclastic activating factor [26], which promoted bone resorption directly. The frequency of bone involvement was between 3% and 13%, according to a large retrospective survey [5]. With the increasing application of magnetic resonance imaging and nuclear imaging, bone involvement is now observed in almost one third of all patients with sarcoidosis [19]. Pain, swelling and numbness are the most common symptoms in patients with bone involvement, whereas almost one half of patients are asymptomatic when diagnosed with bone sarcoidosis [16,18]. All bones could be affected during the process of sarcoidosis. The axial skeleton, for example, as well as the spine and pelvis, are the most commonly affected bones [16,18]. The different sites of bone involvement show different radiographic patterns. For example, the phalanxes of the hands and feet show lytic lesions, while sclerotic lesions occur in spine involvement [27]. Sometimes sarcoidosis might cause malignancy in the bones; in these cases, biopsy is needed for differentiation from other bone disorders [28]. Sarcoidosis patients with bone involvement are more likely to suffer from multiple organ involvement (>3 organs) and tend to receive Infliximab treatment when compared with those without bone involvement [16]. The peculiarity of our case was that the patient suffered from a severe bone lesion, which finally led to amputation of her finger. This may have been attributed to the lack of treatment over a long period of time. If the patient had received corticosteroid treatment as the symptoms appeared, the amputation may have been avoided.

Sinonasal involvement is also an uncommon manifestation in patients with sarcoidosis; its frequency is reported to be 1–6.5% [6,7]. The mucosa, nasal bones or paranasal sinuses can be affected during the disease, and the symptoms are diverse according to the status of the affected sites. The characteristics of sinonasal sarcoidosis in previous studies are shown in Table 3 [6,7,29,30,31,32,33]. Chronic rhinitis, nasal obstruction and anosmia were the most common symptoms in sinonasal sarcoidosis [34]. Lawson et al. divided sinonasal sarcoidosis into four subtypes (hypertrophic, atrophic, destructive, and nasal enlargement) according to specific manifestations [30]. The hypertrophic subtype manifests as diffused extravasated blood in the mucosa accompanied by secondary nasal infection; furthermore, patients may have rhinosinusitis-like symptoms and polyp formation. Patients with the atrophic subtype might experience crusting and nosebleeds due to erosion of their nasal mucosa. The destructive subtype presents with damage to the skeletal structure of the nasal bones, including the nasal septum, lateral nasal walls and turbinates; these conditions might lead to the formation of a fistula. The nasal enlargement subtype refers to the elevation of external nose size which is reversible after corticosteroid treatment. Krespi et al. proposed a staging system which categorized patients according to the extent of disease and the efficacy of corticosteroid therapy [35]. Local steroid sprays, intralesional steroid injections, or systemic corticosteroids can be applied for the therapy of sinonasal sarcoidosis, and surgical treatment could be effective for those who fail to respond to corticosteroid treatment [35]. Our patient experienced swelling of the mucosa, destruction of the nasal bone and enlargement of the inferior turbinate. Fortunately, she received corticosteroid treatment in a timely manner and these symptoms largely improved; this avoided nasal surgery.

## 4. Conclusions

Here, we report a patient with sarcoidosis who mainly presented with severe bone and sinonasal involvement. The patient experienced enlargement of the intrathoracic lymph nodes, swelling of right middle finger and nasal obstructive for several years. The lack of corticosteroid therapy at an early stage ultimately resulted in amputation of the right middle finger. This case highlights the differential diagnosis of systemic granulomatous diseases and indicates the necessary for corticosteroid therapy when treating systematic sarcoidosis.

## Figures and Tables

**Table 1:** Laboratory test results and reference ranges.

Items	Result	Reference Range
White blood cell	3.61 × 10^9^/L	(3.5–9.5) × 10^9^/L
Hemoglobin	131 g/L	(110–150) g/L
Platelet	239 × 10^9^/L	(125–350) × 10^9^/L
Calcium	2.19 mmol/L	(2.1–2.52) mmol/L
Albumin	38.7 g/L	(40–55) g/L
Aspartate transaminase	16 U/L	(13–35) U/L
Brain natriuretic peptide	34 pg/L	(0–100) pg/L
Creatinine	56 umol/L	(41–81) umol/L
Urea	7.00 mmol/L	(3.10–8.80) mmol/L
C-reactive protein	8 mg/L	(0–8) mg/L
Erythrocyte sedimentation rate	8 mm/h	(0–20) mm/h
PPD	Negative	
T-SPOT	Negative	
Glucatell test	Negative	
Glactomannan test	Negative	
Cryptococcus antigen test	Negative	
Rheumatoid antibody	Negative	
Anti-nuclear antibody	Negative	
Anti-double stranded DNA antibody	Negative	
Anti-Smith antibody	Negative	
Immunofixation electrophoresis	Negative	
Anti-neutrophil cytoplasmic antibodies	Negative	
IgG4	0.285 g/L	(0.030–2.010) g/L
Ferritin	152 μg/L	(13–150) μg/L
Urinal red blood cells	2.67/HPF	(0–2.5)/HPF
Urinal white blood cell	4.04/HPF	(0.11–2.83)/HPF
Urinal calcium for 24 h	3.54 mmol	(2.7–7.5) mmol

**Table 2:** Bone involvement of sarcoidosis.

Studies	Numbers of Patients with Bone Involvement	Prevalence of Bone Involvement	Age (Median, Range)	The Female:Male Ratio	Numbers of Patients with Bone Symptoms (%)	Sites of Bone Involvement (%)	Image Modalitied (n)
Zhou et al., 2017 [16]	64	3.4%	46.5 (20–68)	1.46:1	36 (56.3%)	Spine (68.8%) Pelvis (35.9%) Hands (15.6%) Femur (14.1%) Rib (10.9%)	MRI (36) PET-CT (32) CT (14) X-ray (8)
Papiris et al., 2020 [17]	29	4.9%	NA	NA	NA	Femur (37.9%) Spine (31%) Ribs (24.1%)	PET/CT (29)
Sparks et al., 2014 [18]	20	1.5%	51 (40–74)	1.2:1	10 (50%)	Spine (70%) Pelvis (65%) Scapula (35%) Sternum (15%) Femur (30%) Humerus (25%) Phalanges (10%)	MRI (13) PET-CT (9) CT (4) X-ray (2)
Mostard et al., 2012 [19]	32	34%	42 (26–65)	1.9:1	NA	Spine (47%) Pelvis (40%) Ribs (18%) Extremities (34%)	PET-CT (32)
Grozdic et al., 2016 [20]	18	22%	48 (33–72)	1:1	10 (55%)	Pelvis (61.1%) Spine (44.4%) Ribs (27.8%)	PET-CT (18)
Demaria et al., 2020 [21]	12	14%	51.3 (35–75)	1:1	5 (42%)	Spine (91.7%) Pelvis (66.7%) Sternum (33.3%) Humerus (25%) Femur (16.7%)	PET-CT (12)
Neville et al., 1976 [22]	24	NA	NA	2:1	14 (58%)	Hands and/or feet (83.3%)	X-ray (24)

NA: Not applicable.

**Table 3:** Sinonasal involvement of sarcoidosis.

Studies	Numbers of Patients with Sinonasal Involvement	Prevalence of Sinonasal Involvement	Age (Median, Range)	Female:Male Ratio	Symptoms (%)	Manifestations in Endoscopy Examination (%)
Aubart et al., 2006 [7]	20	1.6%	32 (23–41)	1.86:1	Obstruction (90%) Rhinorrhea (70%) Anosmia (70%) Crusting (55%) Epistaxis (30%)	Mucosal hypertrophy (75%) Granular mucosa (50%) Nasal polyps (30%) Septal perforation (5%)
Braun et al., 2004 [6]	15	NA	44 (31–73)	1.1:1	Obstruction (80%) Rhinorrhea (60%) Crusting (40%) Epistaxis (40%) Anosmia (13.3%)	NA
Fergie et al., 1999 [29]	8	NA	44 (21–79)	3:1	Obstruction (87.5%) Epiphora (50%) Rhinorrhea (25%) Anosmia (12.5%) Epistaxis (12.5%)	Mucosal hypertrophy (25%) Congested mucosa (25%) Crusting (37.5%) Nasal polyps (25%) Granular mucosa (25%)
Lawson et al., 2014 [30]	14	NA	42 (29–63)	2.5:1	Rhinorrhea (57.1%) Obstruction (42.9%) Crusting (42.9%)	Crusting (57.1%) Mucosal hypertrophy (28.6%) Septal perforation (28.6%)
Braun et al., 2010 [31]	22	NA	50 (41–79)	0.8:1	Obstruction (81.8%) Rhinorrhea (72.7%) Crusting (40.9%) Epistaxis (40.9%) Anosmia (40.9%)	Granular mucosa (77.3%) Inflammatory mucosa (68.2%) Nasal crusting (45.5%)
Aloulah et al., 2013 [32]	38	NA	52.5 (NA)	2.8:1	Obstruction (65.8%) Crusting (29.9%) Rhinorrhea (26.3%) Dysphagia (26.3%) Epistaxis (18.4%)	Nasal crusting (55.3%) Mucosal hypertrophy (44.7%) Granular mucosa (21%) Septal perforation (5.3%)
Wilson et al., 1988 [33]	27	3.6%	41 (22–63)	1.1:1	Obstruction (88.9%) Crusting (63.0%) Epistaxis (37.0%) Rhinorrhea (29.6%) Facial pain (22.2%)	NA

NA: Not applicable.

## Data Availability

All data analyzed in this study are included in the published article.
